# Exposure to benzyl butyl phthalate (BBP) leads to increased double-strand break formation and germline dysfunction in *Caenorhabditis elegans*

**DOI:** 10.1371/journal.pgen.1011434

**Published:** 2024-10-24

**Authors:** Ayana L. Henderson, Rajendiran Karthikraj, Emma L. Berdan, Shannan Ho Sui, Kurunthachalam Kannan, Monica P. Colaiácovo

**Affiliations:** 1 Department of Genetics, Blavatnik Institute, Harvard Medical School, Boston, Massachusetts, United States of America; 2 Wadsworth Center, New York State Department of Health, Empire State Plaza, Albany, New York, United States of America; 3 Bioinformatics Core, Harvard T.H. Chan School of Public Health, Boston, Massachusetts, United States of America; 4 Department of Environmental Health Sciences, School of Public Health, University at Albany, State University of New York, Albany, New York, United States of America; Stowers Institute for Medical Research, UNITED STATES OF AMERICA

## Abstract

Benzyl butyl phthalate (BBP), a plasticizer found in a wide range of consumer products including vinyl flooring, carpet backing, food packaging, personal care products, and children’s toys, is an endocrine-disrupting chemical linked to impaired reproduction and development in humans. Despite evidence that BBP exposure perturbs the integrity of male and female gametes, its direct effect on early meiotic events is understudied. Here, using the nematode *Caenorhabditis elegans*, we show that BBP exposure elicits a non-monotonic dose response on the rate of X-chromosome nondisjunction measured using a high-throughput screening platform. From among the range of doses tested (1, 10, 100 and 500 μM BBP), we found that 10 μM BBP elicited the strongest effect on the germline, resulting in increased germ cell apoptosis and chromosome organization defects. Mass spectrometry analysis shows that *C*. *elegans* efficiently metabolizes BBP into its primary metabolites, monobutyl phthalate (MBP) and monobenzyl phthalate (MBzP), and that the levels of BBP, MBP, and MBzP detected in the worm are within the range detected in human biological samples. Exposure to 10 μM BBP leads to germlines with enlarged mitotic nuclei, altered meiotic progression, activation of a p53/CEP-1-dependent DNA damage checkpoint, increased double-strand break levels throughout the germline, chromosome morphology defects in oocytes at diakinesis, and increased oxidative stress. RNA sequencing analysis indicates that BBP exposure results in the altered expression of genes involved in xenobiotic metabolic processes, extracellular matrix organization, oocyte morphogenesis, meiotic cell cycle, and oxidoreduction. Taken together, we propose that *C*. *elegans* exposure to BBP leads to increased oxidative stress and double-strand break formation, thereby compromising germline genomic integrity and chromosome segregation.

## Author summary

Endocrine disrupting chemicals (EDCs) have been linked to various health problems including effects on reproductive health. Benzyl butyl phthalate (BBP) is a commonly used plasticizer found in many consumer products that has been shown to act as an EDC and affect human reproduction and development. However, how it might impact the germline and affect the specialized cell division program of meiosis, which results in the formation of haploid gametes (i.e. eggs and sperm), remained unclear. Here, using the nematode *C*. *elegans*, we show that BBP exposure at levels within the range of what is detected in humans impairs accurate chromosome segregation, which can result in aneuploidy. Similar to mammals, BBP exposure in *C. elegans* results in a non-monotonic dose response, whereby lower doses result in the strongest effects, and BBP is efficiently metabolized, underscoring the use of this model system for gaining mechanistic insights into how this EDC impacts the germline. BBP results in increased levels of DNA double-strand breaks (DSBs), activation of a DNA damage checkpoint, and altered chromosome morphology in exposed germlines. We propose that BBP exposure alters gene expression and results in increased oxidative stress, resulting in increased DSBs, thereby compromising chromosome morphology and accurate segregation.

## Introduction

Endocrine disrupting chemicals (EDCs) are found in a plethora of products utilized by humans daily. This underscores our ubiquitous, and often unintentional, exposure to toxicants that have the potential to disrupt various physiological processes such as meiosis. Several studies have highlighted a correlation between EDC exposure and increased incidence of meiotic aneuploidy [1], a phenomenon that underlies a large percentage of reproductive health issues [2,3]. Moreover, studies have linked EDCs to multigenerational and transgenerational effects on reproduction [4]. Although evidence suggests an urgent need to assess EDC-induced changes in the germline, very few studies have addressed the direct effects of EDCs on the meiotic events that take place in the germline.

Phthalates, diesters (dialkyl or alkyl aryl) of ortho-phthalic acid, are one of the best studied classes of EDCs [5]. As plasticizers, they are commonly used to make plastic products more durable and flexible. Benzyl butyl phthalate (BBP) has been found in household maintenance and cleaning reagents, cosmetics, and personal care products, as well as in children’s toys and plastic food packaging [6]. Whether topically applied, sprayed, or consumed, the various uses of BBP-containing products introduce three main routes of exposure for humans: dermal absorption, inhalation, and ingestion. Upon entering the body, BBP is rapidly metabolized by intestinal esterases and lipases as well as by cytochrome P450 in the liver into two primary metabolites: monobutyl phthalate (MBP) and monobenzyl phthalate (MBzP) [7–10]. According to several biomonitoring studies, BBP and its metabolites have been detected in various human biological samples including urine, semen, blood, umbilical cord blood, breast milk, and amniotic fluid [11]. Therefore, exposure to these EDCs may pose a threat to human health, both to those directly exposed as well as to unexposed subsequent generations.

Although its mode of action is not completely understood, BBP has been reported to mimic estrogen and exhibit weak estrogenic activity [12–16]. As a result, exposure to even low doses of BBP has the potential to alter normal hormonal levels and subsequently affect tissue development and reproductive physiology [17]. Epidemiological studies have sought to understand the correlation between internal levels of phthalates and various reproductive outputs. In women, BBP and its metabolites have been correlated with preterm delivery, altered fetal growth, and abnormal development [18]. In men, BBP has been correlated with increased sperm aneuploidy and DNA damage [19]. Given the limitations of assessing the impact of phthalates in human reproductive health, especially in women [20], studies in model organisms have further elucidated the reprotoxic effects of BBP. In female rats, BBP exposure was found to decrease ovarian weight and increase embryonic lethality, preimplantation loss, and fetal defects [21,22]. Recent studies using porcine and mouse oocytes also showed that BBP exposure impaired oocyte meiotic maturation and early embryo development [23,24]. However, while these studies have provided significant insight into the reprotoxic effects of BBP, the effect of BBP on early stages of meiosis remains largely understudied.

The integrity of each step during meiotic prophase I directly influences the function of the germline and the overall quality of gametes. Therefore, any external factor that has the potential to perturb these events can ultimately affect reproductive health. Because assessing how exposures to phthalates may affect meiosis is a daunting task in humans, and often timely and expensive in mammalian models, the use of the nematode *Caenorhabditis* elegans as a model system is highly advantageous for this type of study. *C*. *elegans* is a tractable organism whose meiotic program is very well characterized and shares a high degree of gene conservation with humans [25,26]. Its rapid life cycle coupled with the availability of high-throughput screening methods expedites the identification of chemical exposures affecting meiosis [27,28]. Furthermore, the *C*. *elegans* germline has been shown to provide a sensitive means to detect low EDC exposure effects and to parse out the distinct effects from structural analogs [29].

Here, we assessed the dose response to BBP exposure on X-chromosome nondisjunction, germ cell apoptosis, and germline chromosome morphology in *C*. *elegans*. This revealed that exposure to 10 μM BBP elicited the strongest effect on the germline and showed that worms, like humans, display a non-monotonic dose response to phthalate exposure. Mass spectrometry analysis of the internal levels of BBP and its primary metabolites in the worm following 10 μM BBP exposure identified levels that are comparable to what has been detected in various human biological samples. Subsequent analyses using a 10 μM BBP dose of exposure revealed defects in meiotic progression, as indicated by the presence of nuclei from earlier stages of meiotic prophase I persisting into late pachytene (laggers) and activation of DNA damage checkpoints as suggested by the increased diameter of nuclei in the premeiotic tip and p53/CEP-1-dependent activation of germ cell apoptosis in late pachytene. Immunostaining for the RAD-51 recombinase protein showed that BBP exposure leads to elevated double-strand break (DSB) formation throughout the germline. Increased chromosome morphology defects in oocytes at diakinesis indicate DNA damage persisting in late prophase I. RNA sequencing analysis on whole worm lysates identified 344 differentially expressed genes following BBP exposure. GO term analysis suggests that these genes act in xenobiotic metabolism, extracellular matrix organization, oocyte morphogenesis, oocyte fate determination, meiotic cell cycle regulation, and oxidoreduction. Analysis using an oxidative stress reporter showed increased GFP expression driven by the glutathione S-transferase promoter in the worm germline following BBP exposure. Taken together, our data suggest that exposure to an environmentally-relevant level of BPP results in increased oxidative stress and altered gene expression, compromising germline genomic integrity leading to increased chromosome nondisjunction.

## Results

### BBP exposure elicits non-monotonic dose responses on X-chromosome segregation, germ cell apoptosis, and germline chromosome morphology

**Fig 1:**
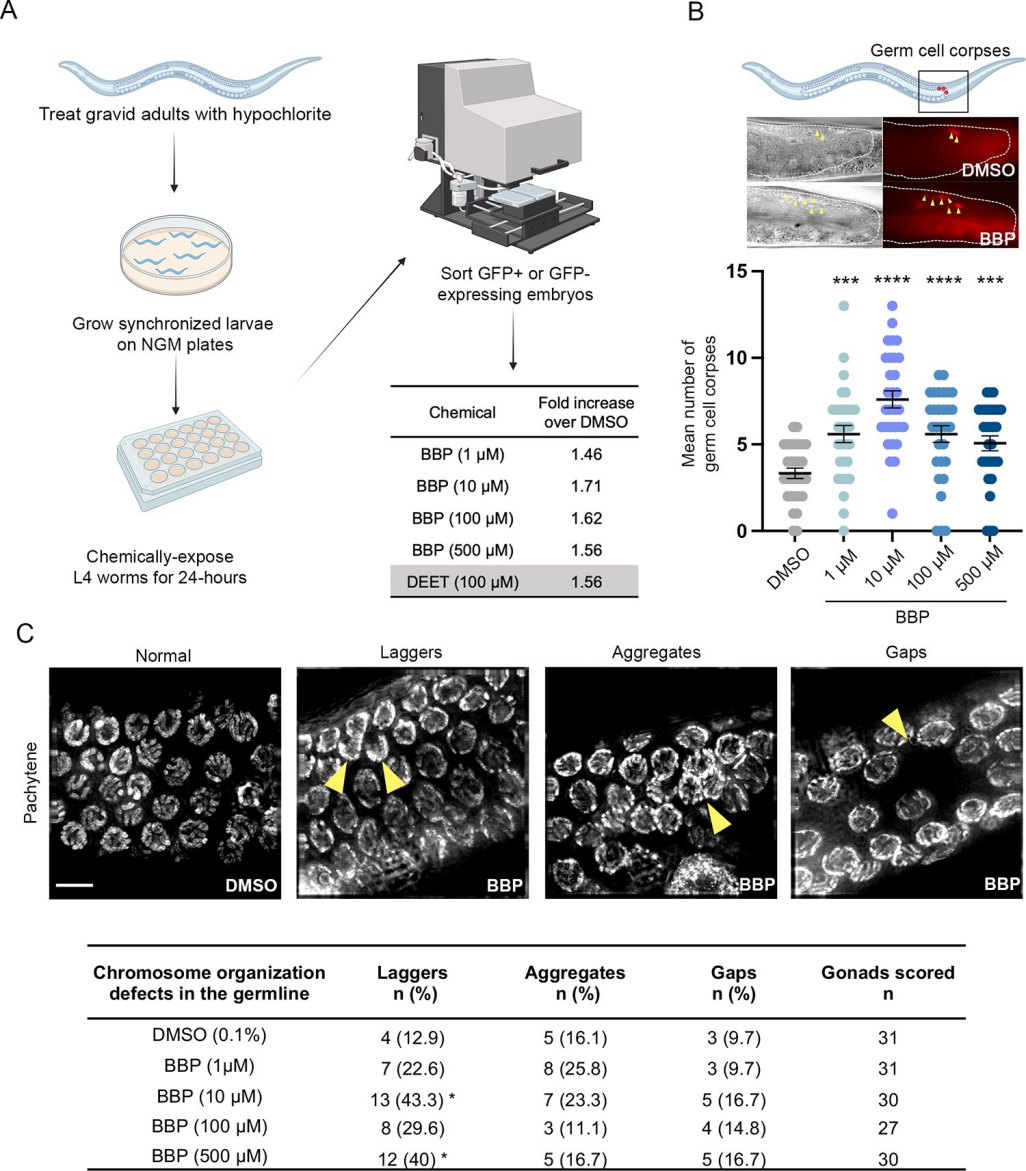
BBP dose-response curve reveals non-monotonic effects of BBP on rate of X-chromosome nondisjunction, apoptosis, and chromosome organization defects in the germline. **(A)** The experimental workflow for high-throughput screening of X-chromosome nondisjunction. *Pxol-1*::*gfp;col-121* worms were synchronized by hypochlorite treatment, grown to the L4 larval stage on NGM plates, and exposed on multi-well plates for 24 hours to 1, 10, 100, and 500 μM of BBP or DMSO (0.1%) vehicle control. Approximately 4,000 worms were screened in two biological repeats for each condition. Gated adults were sorted by GFP expression in embryos *in utero*. Frequency of X-chromosome nondisjunction in BBP-exposed worms was quantified as fold increase over DMSO. Gray bar indicates exposure to 100 μM DEET, an internal positive control. Diagram created with BioRender.com. **(B)** Schematic of *C*. *elegans* highlighting late pachytene as the stage where germ cell apoptosis was assessed. Representative images of acridine orange-stained germ cell corpses in late pachytene of DMSO or BBP-exposed worms in DIC (left) and TRITC (right). Arrowheads indicate germ cell corpses. Quantification of the mean number of germ cell corpses observed following exposure to each chemical condition. Error bars represent SEM. ***P<0.001, ****P<0.0001 by the two-tailed Mann-Whitney test, 95% C.I. N = 30 per condition. Four biological repeats. Diagram created with BioRender.com. **(C)** Carnoy’s fixed and DAPI-stained images of gonads at the pachytene stage following exposure to DMSO or BBP. Images represent examples of gonads with normal germline configuration (first panel) or various chromosome organization defects in the germline including laggers (second panel), aggregates (third panel), and gaps (fourth panel). Yellow arrowheads indicate the respective defect in each panel. Quantification of chromosome organization defects below. *P = 0.0106 for 10 μM BBP, *P = 0.0212 for 500μM BBP, Fisher’s exact test, 95% C.I. N = 27–31 gonads. Three biological repeats. Scale bar, 5 μm.

We previously found, when assessing a panel of common environmental chemicals using a high-throughput screening platform in *C*. *elegans*, that exposure to 100 μM of BBP induces X-chromosome nondisjunction [28]. Given the non-monotonic nature of phthalates [17], we applied the same high-throughput method to screen various doses of BBP and identify the lowest dose that elicits the strongest rate of X-chromosome nondisjunction in *C*. *elegans*. We used worms carrying *col-121(nx3)*, a collagen gene mutation, to increase cuticle permeability and enable analysis of lower doses of exposure, and *Pxol-1*::*gfp*, a transcriptional reporter with GFP expression under the control of a male-specific promoter allowing the detection of GFP+ embryos in utero that are destined to become male (XO) progeny. Taking advantage of the fact that *C*. *elegans* are transparent, we used a large object flow cytometry system (COPAS Biosort; Union Biometrica) to rapidly sort worms based on fluorescence intensity. *Pxol-1*::*gfp; col-121* hermaphrodites were first age-matched by hypochlorite treatment, raised to the late larval 4 (L4) stage, and exposed to 1, 10, 100, and 500 μM of BBP or DMSO (0.1%) vehicle control for 24 hours to assess the effect of exposure during oogenesis. As a positive control, worms were exposed to 100 μM of DEET, an insect repellent recently reported to cause increased X chromosome nondisjunction in *C*. *elegans* [30]. Approximately 4,000 worms were screened in two biological repeats for each condition. We found that exposure to 10 μM of BBP resulted in the strongest increase in X nondisjunction relative to DMSO vehicle control (1.71-fold; **Fig 1A**). When examining the progeny laid on plates by individual exposed worms, this dose also resulted both in increased embryonic lethality and male progeny (P<0.0001 by the Fisher’s exact test), which is suggestive of autosomal and X chromosome nondisjunction, respectively (**S1 Fig**). To further define the lowest dose of BBP exposure with the strongest effects in the germline, we assessed germ cell apoptosis by acridine orange staining [31] in *col-121* worms exposed to 1, 10, 100, and 500 μM of BBP or DMSO (0.1%) vehicle control for 24 hours. BBP exposure led to significant increase in the mean number of germ cell corpses in a non-monotonic manner whereby 10 μM exhibited the strongest effects on germ cell apoptosis relative to other doses (****P<0.0001 by the two-tailed Mann-Whitney test, 95% C.I.; **Fig 1B**). Finally, we examined chromosome morphology and organization throughout the germline at all four doses of BBP exposure compared to vehicle alone. Exposures to 1 and 100 μM BBP did not show significant differences in the number of gonads with leptotene/zygotene-like nuclei persisting into the pachytene stage (laggers), nuclei forming aggregates, and areas with a reduced density of nuclei (gaps). However, worms exposed to 10 μM and 500 μM of BBP exhibited significant increases in the frequency of laggers (*P = 0.0106 and *P = 0.0212, respectively, by the Fisher’s exact test, 95% C.I.), and in the total number of defects combined (83.3% and 73.3%, respectively, compared to 38.7% in vehicle alone; ***P<0.001 for 10 μM BBP and **P<0.01 for 500 μM BBP; **Fig 1C**). Taken together, these three combined approaches revealed that exposure to 10 μM BBP results in the strongest effects on the germline, and that BBP also elicits a non-monotonic dose response in *C*. *elegans*.

### Mass spectrometry reveals internal levels of BBP and its primary metabolites within the worm

**Fig 2:**
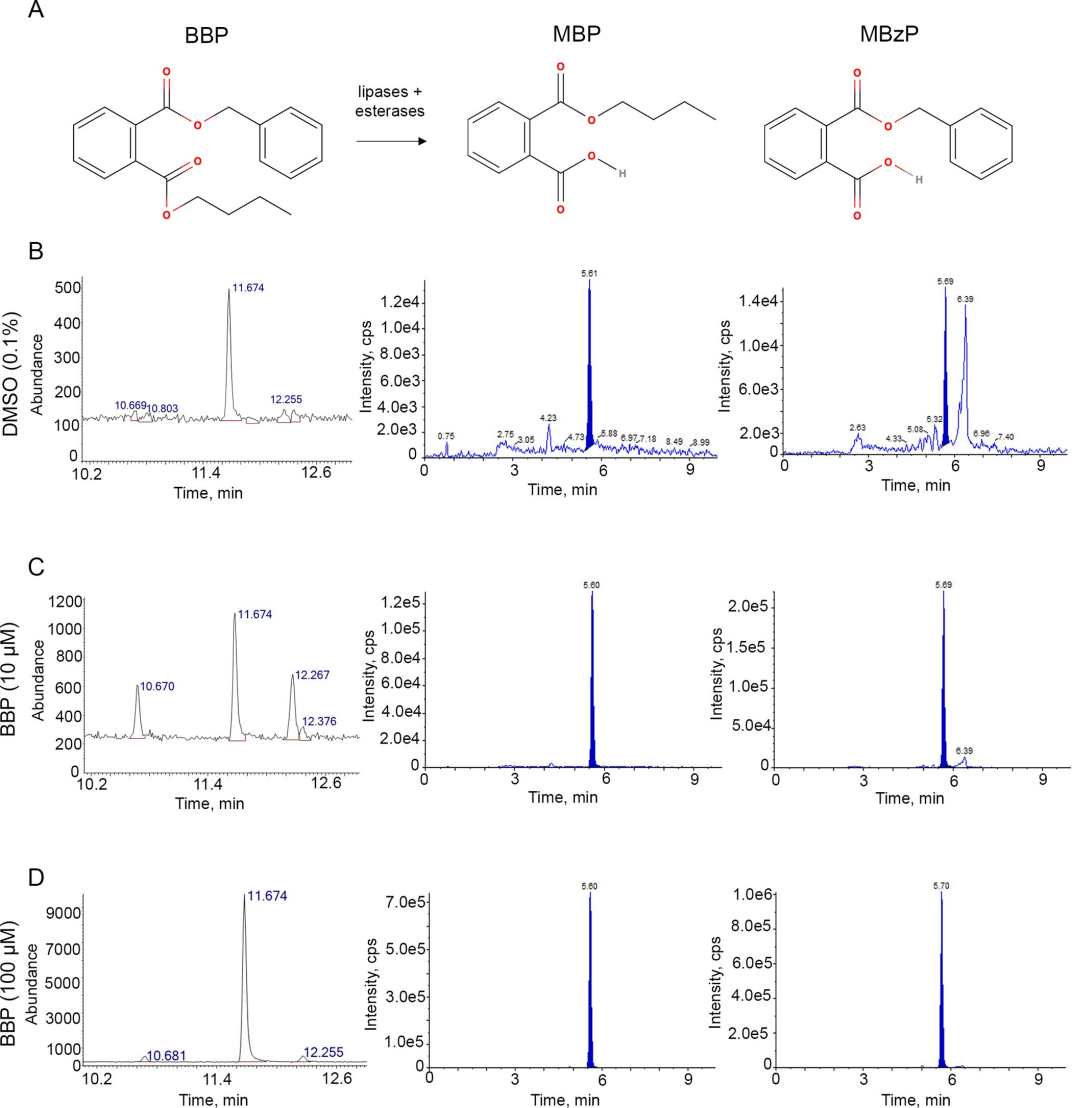
Mass spectrometry reveals physiologically relevant, internal levels of BBP and its metabolites within the worm. **(A)** BBP is metabolized by lipases and esterases into its two primary metabolites: MBP and MBzP. Gas chromatography mass spectrometry was used to measure BBP, while liquid chromatography with tandem mass spectrometry was used to measure MBP and MBzP in the worm following chemical exposure. Chromatograms represent the detected concentrations of BBP (left), MBP (middle), and MBzP (right) following exposure to **(B)** 0.1% DMSO vehicle control, **(C)** 10 μM of BBP, and **(D)** 100 μM of BBP. X-axis represents retention time; y-axis represents abundance or intensity (cps).

In humans, BBP is metabolized by gut esterases and lipases to produce two primary metabolites: mono-n-butyl phthalate (MBP) and mono-n-benzyl phthalate (MBzP) ([9]; **Fig 2A**). Given the results from our dose response studies, we selected two doses, 10 μM and 100 μM, along with DMSO vehicle control to measure internal levels in the worm. Gas chromatography mass spectrometry was used to measure BBP, while liquid chromatography with tandem mass spectrometry was used to measure MBP and MBzP. In response to DMSO exposure, we detected 0.026 μg/mL of BBP, 0.003 μg/mL of MBP, and 0.002 μg/mL of MBzP in our worm lysates **(Fig 2B)**. In response to 10 μM of BBP exposure, we detected 0.083 μg/mL of BBP, 0.104 μg/mL of MBP, and 0.282 μg/mL of MBzP **(Fig 2C)**. In response to 100 μM of BBP exposure, we detected 0.801 μg/mL of BBP, 0.630 μg/mL of MBP, and 1.508 μg/mL of MBzP **(Fig 2D)**. These data indicate that worms, like humans, can metabolize BBP to its monoester metabolites. Moreover, the internal concentration of BBP and its metabolites detected in the worms exposed to 10 μM BBP were within the range detected in human biological samples (e.g., urine and serum). The internal concentration of BBP detected in cord blood (at the 95^th^ percentile) was 0.089 μg/mL [18], while up to 0.336 μg/mL of MBzP has been detected in human maternal urine samples [32], and up to 0.263 μg/mL of MBP in human amniotic fluid [33]. Given that exposure to 10 μM BBP impacts the worm germline and results in internal levels within the range of what is found in human biological samples, all subsequent experiments were conducted at this dose.

### BBP alters meiotic progression and induces p53-dependent DNA damage checkpoint activation

**Fig 3:**
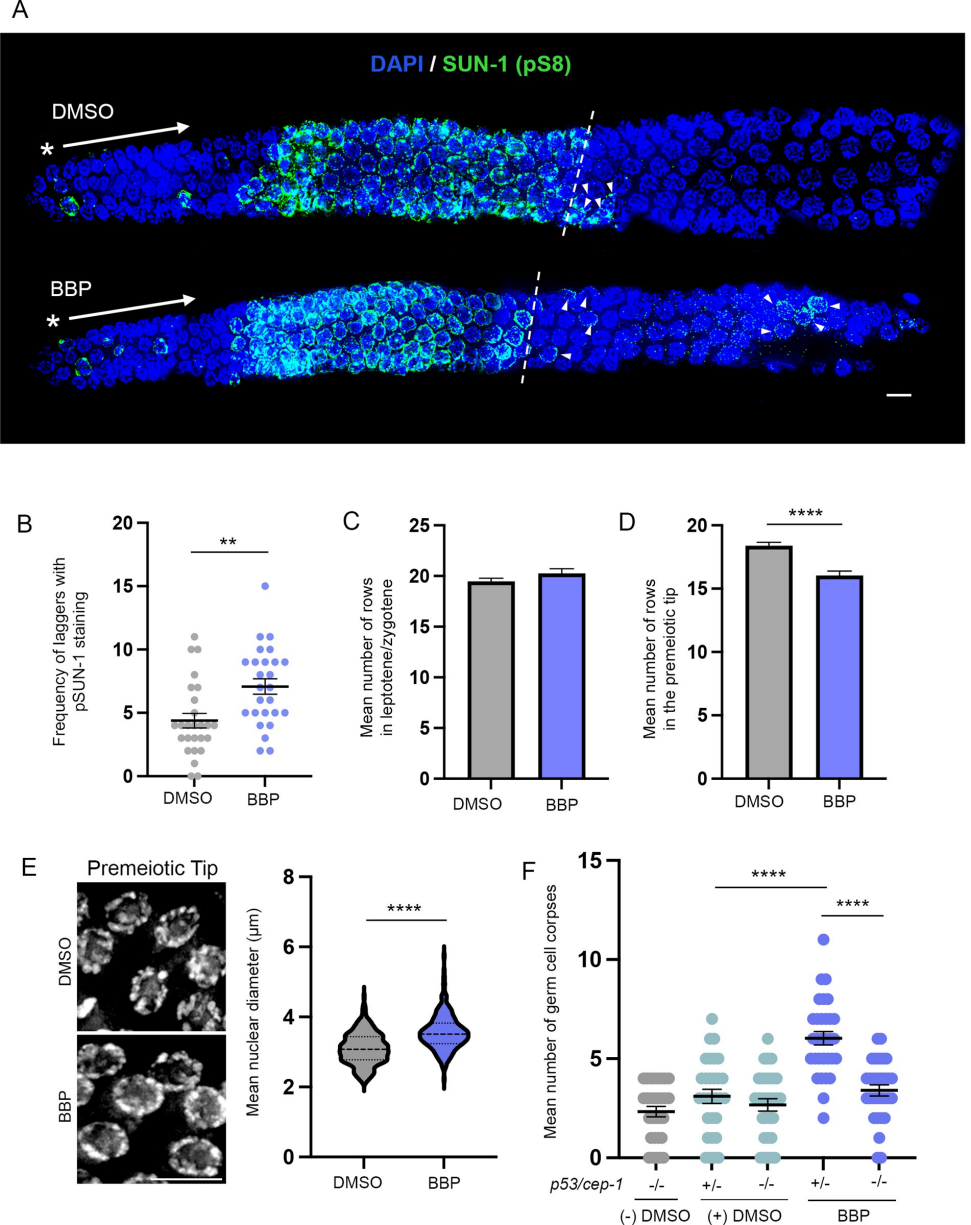
BBP alters meiotic progression and leads to activation of a DNA damage checkpoint. **(A)** High-resolution images of whole mounted dissected gonads stained with DAPI (blue) and immunostained against SUN-1 (pS8) (green) following DMSO or BBP exposure. Asterisks indicate the orientation of the gonad, starting from the premeiotic tip to late pachytene. Dashed lines mark the end of the rows with all nuclei showing SUN-1 (pS8) signal (end of the leptotene/zygotene stage). Arrowheads indicate “laggers”, which are pS8^+^ nuclei persisting into pachytene. Scale bar, 5 μm. **(B)** Quantification of the frequency of laggers detected per gonad with pSUN-1 staining in worms of the indicated chemical treatment. Error bars represent SEM. **P<0.01 by the two-tailed Mann-Whitney test, 95% C.I. N = 26 gonads per condition. Five biological repeats. **(C)** Quantification of the mean number of rows of nuclei in leptotene/zygotene following DMSO or BBP exposure. Error bars represent SEM. N = 30 gonads per condition. Three biological repeats. **(D)** Quantification of the mean number of rows of nuclei in the premeiotic tip following DMSO or BBP exposure. Error bars represent SEM. ****P<0.0001 by the two-tailed Mann-Whitney test, 95% C.I. N = 30 gonads per condition. Three biological repeats. **(E)** Left, high magnification representative images of nuclei in the premeiotic tip from hermaphrodites exposed to DMSO or BBP. Scale bar, 5 μm. Right, quantification of the mean nuclear diameter (μm) in worms following DMSO or BBP exposure. ****P<0.0001 by the two-tailed Mann-Whitney test, 95% C.I. N ≥ 211 nuclei from 6 gonads per condition. Two biological repeats. **(F)** Quantification of the mean number of germ cell corpses in *cep-1 (-/-)* or *(+/-)* hermaphrodites either unexposed, exposed to DMSO vehicle control, or exposed to 10 μM of BBP. Error bars represent SEM. ****P<0.0001 by the two-tailed Mann-Whitney test, 95% C.I. N = 30–31 gonads per condition. Four biological repeats.

Given the chromosome morphology defects observed throughout the germline, we further assessed the effects of BBP on meiotic progression. During the leptotene/zygotene stage, SUN/KASH domain-containing proteins form complexes bridging the nuclear envelope (NE) that tether a single end of each chromosome to the NE allowing for the transmission of cytoplasmic cytoskeletal forces through the NE that drive the movement and pairing of homologous chromosomes [34]. To examine meiotic progression, we assessed the localization of phosphorylated SUN-1 (pS8), which in wild type is detected as bright aggregates at the NE during leptotene/zygotene that then decrease in intensity and disperse upon entrance into pachytene [35]. We found that while the overall length of the zone with leptotene/zygotene stage nuclei is comparable between DMSO and BBP-exposed worms **(Fig 3A and 3C)**, the frequency of pS8^+^ nuclei detected at later stages of pachytene (laggers) is significantly increased in BBP-exposed germlines (**P<0.01 by the two-tailed Mann-Whitney test, 95% C.I.; **Fig 3B**), indicating defects in meiotic progression. Impaired meiotic progression can result from stalled premeiotic or meiotic events in response to checkpoint activation [36]. To examine mitotic checkpoint activation, we measured the number of rows of nuclei as well as the diameter of nuclei specifically in the premeiotic tip region of the germline given that fewer numbers of nuclei and an increased diameter are indicative of stress-induced replication-dependent S-phase checkpoint activation [37,38]. BBP exposure led to a significant decrease in the mean number of rows of nuclei in the premeiotic tip (****P<0.0001; two-tailed Mann-Whitney test, 95% C.I.), and a significant increase in mean nuclear diameter (****P<0.0001; two-tailed Mann-Whitney test, 95% C.I.), suggesting activation of a checkpoint **(Fig 3D and 3E)**. A p53/CEP-1-dependent checkpoint has been shown to be activated in response to DNA damage resulting in the induction of germ cell apoptosis in late pachytene [39,40]. Analysis by acridine orange staining revealed a p53/CEP-1-dependent increase in germ cell apoptosis in the BBP-treated worms **(Fig 3F)**. We found that while BBP exposure in the *p53/cep-1* (+/-) background leads to a significant increase in the mean number of germ cell corpses (****P<0.0001 by the two-tailed Mann-Whitney test, 95% C.I.), that effect is lost in the *p53/cep-1* (-/-) background, supporting a p53-dependent checkpoint activation in response to BBP exposure. Therefore, our data demonstrate BBP-induced alterations in meiotic progression coupled with potential checkpoint activation at the premeiotic tip region and p53/CEP-1-dependent checkpoint activation in late meiotic prophase.

### BBP leads to increased mitotic and meiotic DSB formation and defects in oocytes at diakinesis

**Fig 4:**
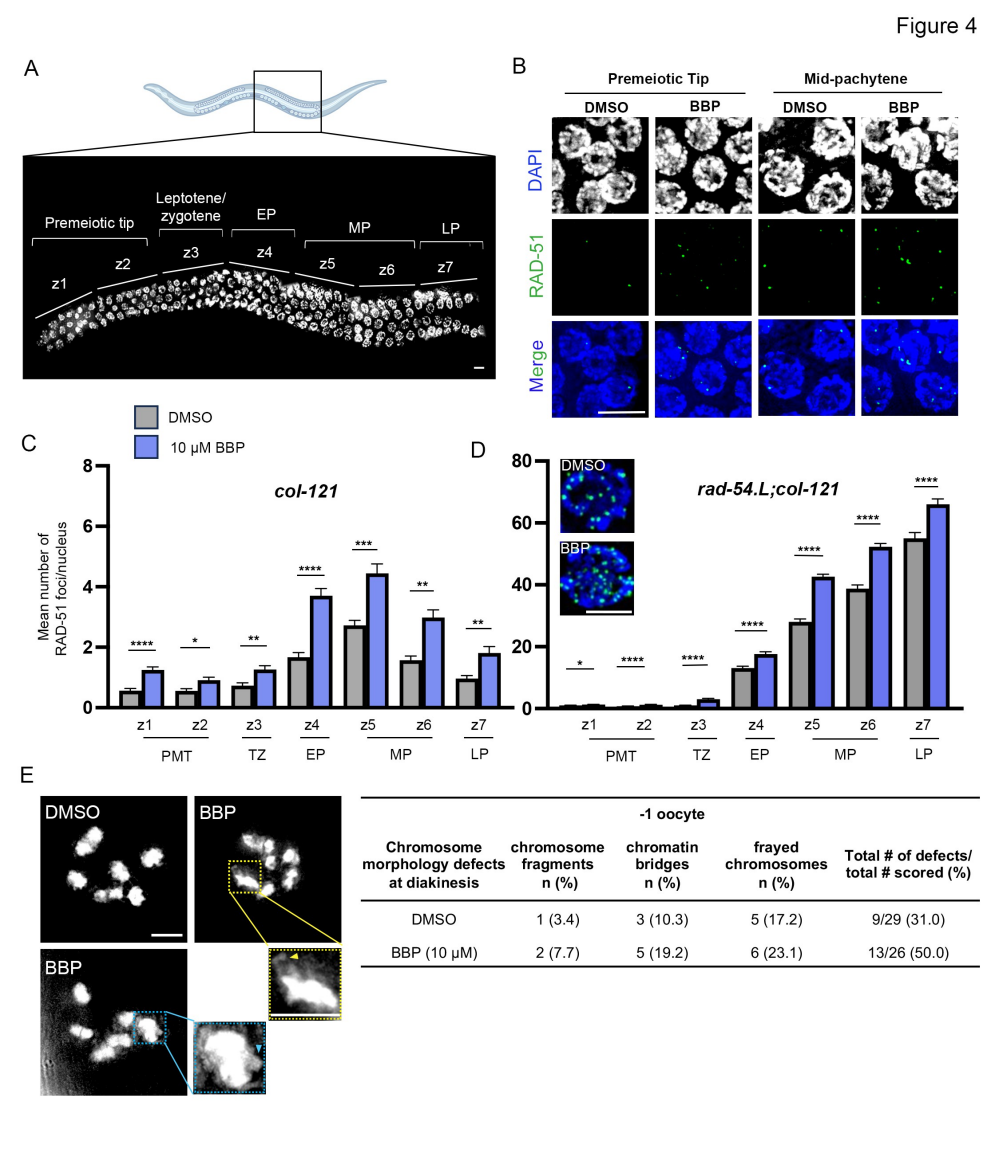
Increased DSB formation throughout the germline and chromosome morphology defects at diakinesis in BBP-exposed worms. **(A)** High-resolution image of a whole-mounted and DAPI-stained *C*. *elegans* hermaphrodite gonad. Zones 1 and 2 correspond to the premeiotic tip; zone 3, leptotene/zygotene; zone 4, early pachytene; zones 5 and 6, mid-pachytene; and zone 7, late pachytene. Scale bar, 5 μm. Diagram created with BioRender.com. **(B)** Representative images of premeiotic tip and mid-pachytene nuclei from hermaphrodite gonads exposed to either DMSO or BBP co-stained with DAPI (blue) and RAD-51 (green). Scale bar, 5 μm. **(C)** Quantification of the mean number of RAD-51 foci per nucleus in each zone of the germlines from DMSO- or BBP-exposed *col-121* worms. Error bars represent SEM. *P<0.05, **P<0.01, ***P<0.001, ****P<0.0001 by the two-tailed Mann-Whitney test, 95% C.I. N = 5 gonads per condition. Two biological repeats. PMT, premeiotic tip; TZ, transition zone (leptotene/zygotene); EP, early pachytene; MP, mid-pachytene; LP, late pachytene. **(D)** Quantification of the mean number of RAD-51 foci per nucleus in each zone of the germlines from DMSO- or BBP-exposed *rad-54*.*L;col-121* worms. Error bars represent SEM. *P<0.05, ****P<0.0001 by the two-tailed Mann-Whitney test, 95% C.I. N = 6 gonads per condition. Two biological repeats. PMT, premeiotic tip; TZ, transition zone (leptotene/zygotene); EP, early pachytene; MP, mid-pachytene; LP, late pachytene. Insets are representative high magnification images of nuclei in mid-pachytene from *rad-54*.*L;col-121* hermaphrodites exposed to DMSO or BBP co-stained with DAPI (blue) and RAD-51 (green). Scale bar, 3 μm. **(E)** High-resolution images of -1 oocytes at diakinesis from DMSO- or BBP-exposed hermaphrodites. Insets shown to facilitate visualization of a chromosome fragment (blue arrowhead) and a frayed chromosome (yellow arrowhead) defect. Quantification of the chromosome morphology defects observed in -1 oocytes at diakinesis including chromosome fragments, chromatin bridges, and frayed chromosomes per chemical condition. For total number of defects scored, P = 0.27 by the Fisher’s exact test, 95% C.I. N = 26–29 gonads per condition. Three biological repeats. Scale bar, 5 μm.

To determine whether some of the chromosome morphology defects detected throughout germline nuclei and the increased p53-dependent germ cell apoptosis reflect problems with DSB formation and repair, we quantified the levels of foci per nucleus for the strand invasion/exchange DSB repair protein RAD-51 [41], throughout seven zones along the germline as in [42] **(Fig 4A)**. This revealed a significant increase in RAD-51 foci throughout the germline, including both the premeiotic and meiotic zones **(Fig 4B and 4C)**. An increase in RAD-51 foci can be a result of either an increase in the total number of DSBs formed or an inability to repair DSBs. To distinguish between these two possibilities, we examined the levels of RAD51 foci along the germlines of *rad-54*.*L*;*col-121* mutants in which meiotic DSBs are produced but do not undergo turnover, allowing us to score the total number of DSB repair sites [43]. This analysis revealed a significant increase in RAD-51 foci throughout the germlines of BBP-exposed worms compared to DMSO, supporting an increase in the overall number of DSBs formed following BBP exposure **(Fig 4D)**. The increased levels of DSBs did not affect crossover (CO) designation levels, as determined by quantification of foci for the pro-CO marker COSA-1/CNTD1 in late pachytene nuclei **(S2 Fig)**. As in wild type [44], both DMSO- and BBP-exposed worms exhibited six COSA-1::GFP foci per nucleus. However, whether CO position along the chromosomes and subsequent late prophase I chromosome remodeling are affected, remains to be determined. Analysis of chromosome morphology in oocytes in late diakinesis (-1 oocytes) revealed a 1.6-fold increase in the levels of oocytes with chromosome fragments, chromatin bridges, and frayed chromosomes **(Fig 4E)**. Although not significant (P = 0.27; Fisher’s exact test, 95% C.I.), this increase suggests that the p53-dependent activation of germ cell apoptosis at late pachytene may not be sufficient to remove all nuclei carrying chromosomes with unrepaired DNA damage. Taken together, these data indicate that BBP exposure produces increased numbers of DSBs throughout the germlines of exposed worms.

### BBP causes differential gene expression of genes related to extracellular matrix processes, oxidative stress, and oocyte integrity

**Fig 5:**
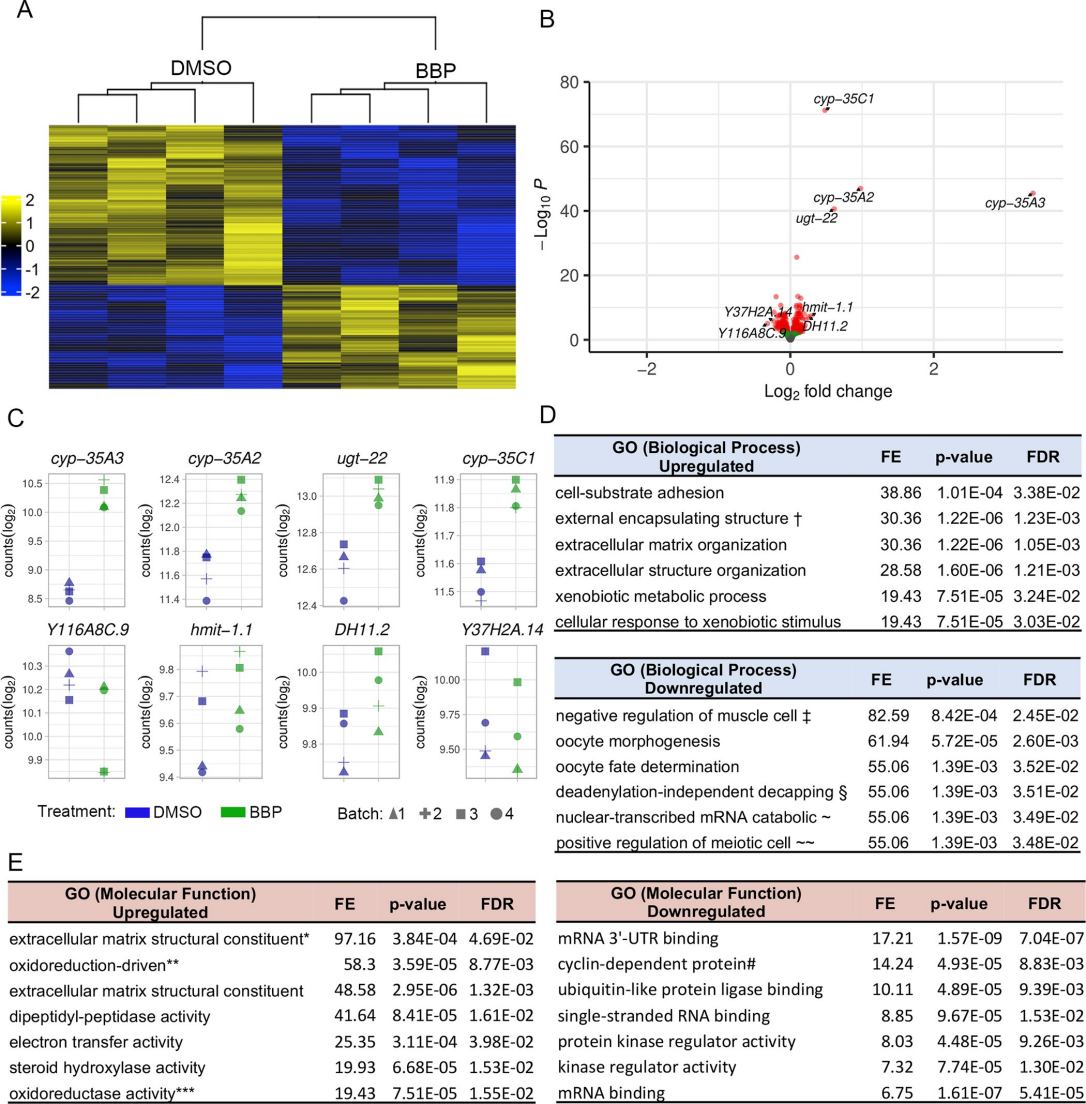
BBP exposure leads to differential gene expression. **(A)** Heatmap of 344 differentially expressed genes identified by RNA-seq from whole worm lysates from DMSO- or BBP-exposed worms (four independent biological repeats). **(B)** Volcano plot highlighting the top 8 differentially expressed genes by effect size in BBP exposed worms compared to DMSO control. **(C)** Top 8 differentially expressed genes by effect size. Blue represents DMSO-exposed worms. Green represents BBP-exposed worms. Symbols indicate the batch or biological repeat. **(D)** Gene ontology enrichment analysis for biological processes in the upregulated gene population (top) or downregulated gene population (bottom). †External encapsulating structure organization; ‡negative regulation of muscle cell differentiation; §deadenylation-independent decapping of nuclear-transcribed mRNA; ~nuclear-transcribed mRNA catabolic process; ~~positive regulation of meiotic cell cycle process. **(E)** GO enrichment analysis for molecular functions in the upregulated gene population (left) or downregulated gene population (right). *Extracellular matrix structural constituent conferring tensile strength; **oxidoreduction-driven active transmembrane transporter activity; ***oxidoreductase activity, acting on paired donors, with incorporation or reduction of molecular oxygen, reduced flavin or flavoprotein as one donor, and incorporation of one atom of oxygen; #cyclin-dependent protein serine/threonine kinase regulator activity. FE, fold enrichment; FDR, false discovery rate.

**Fig 6:**
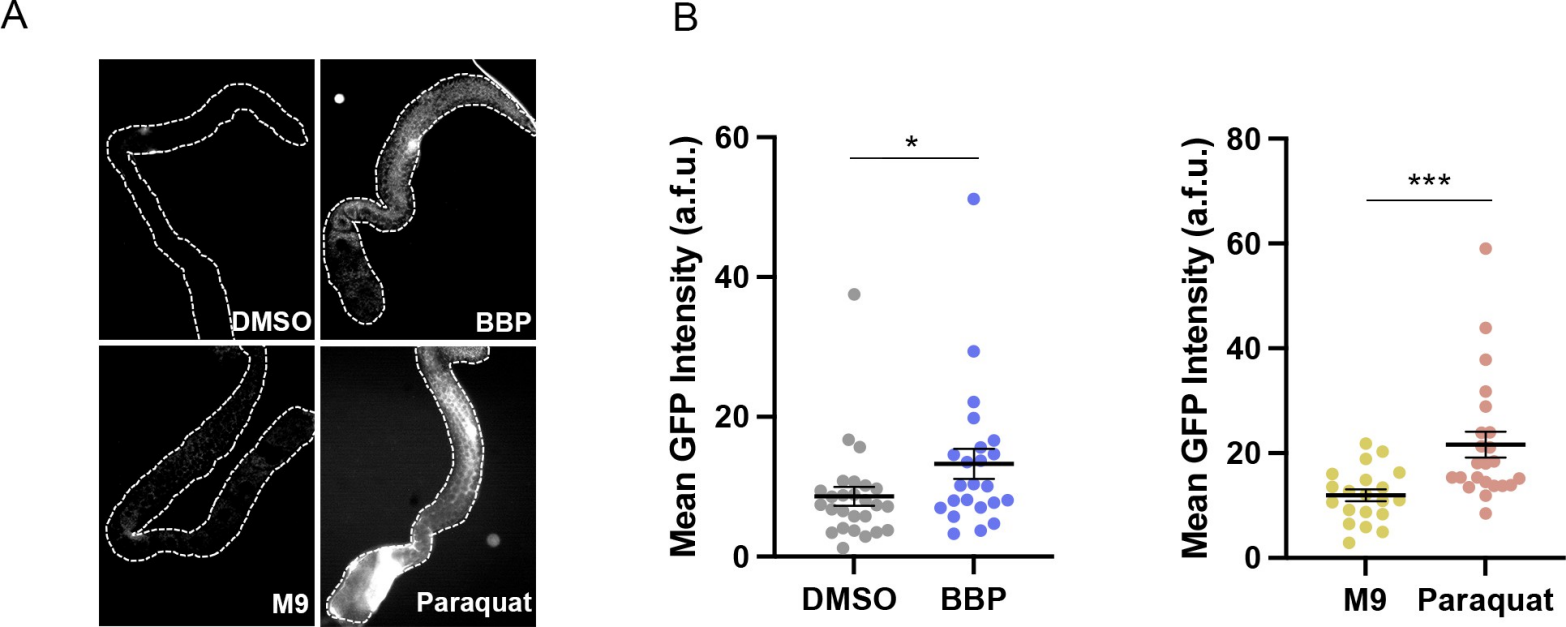
BBP induces oxidative stress within the germline. **(A)** Dissected gonads of *gst-4p*::*gfp;col-121* worms exposed to DMSO, BBP, M9, or Paraquat. Dashed line used to outline the dissected gonads. **(B)** Quantification of the mean GFP intensity (a.f.u., arbitrary fluorescence units) measured in the gonads of *gst-4p*::*gfp;col-121* exposed to DMSO, BBP, M9, or Paraquat. Error bars represent SEM. *P = 0.0392, ***P = 0.0003 by the two-tailed Mann-Whitney test, 95% C.I. N = 20–26 gonads per condition. Four biological repeats.

To gain more insight into the potential mechanisms underlying the increased DSB levels observed throughout the germline of BBP-exposed worms, we assessed the effects of BBP on gene expression. We performed RNA sequencing (RNA-seq) in whole *col-121* worm samples exposed to either 10μM BBP or DMSO for 24-hours (four biological repeats). BBP exposure resulted in the statistically significant differential expression of 344 genes, 103 upregulated and 241 downregulated, which exhibit clear clustering as shown in a heatmap **(Fig 5A)**. Among the top 8 differentially expressed genes by effect size **(Fig 5B and 5C)** are three cytochrome p450 genes, *cyp-35A3*, *cyp-35A2*, and *cyp-35C1*, which are known to play a role in xenobiotic metabolism [45]; *ugt-22*, which encodes for an enzyme that plays a role in UDP-glucuronosyltransferase activity which is critical in phase II drug metabolism [45]; *hmit-1*.*1*, which is predicted to be involved in myo-inositol transport and transmembrane transport and was reported to play a role in various metabolic pathways [46]; and three not fully characterized genes, *Y116A8C*.*9*, *DH11*.*2*, and *Y37H2A*.*14*, which are predicted to encode for a transmembrane protein, a body wall muscle protein, and a *C*. *elegans*-specific protein, respectively [47]. We next applied gene ontology (GO) enrichment analysis to categorize key biological processes among the upregulated and downregulated gene populations by fold enrichment. The top biological processes identified among the upregulated genes include cell-substrate adhesion (GO:0031589), external encapsulating structure organization (GO:0045229), extracellular matrix organization (GO:0030198), extracellular structure organization (GO:0043062), xenobiotic metabolic process (GO:0006805), and cellular response to xenobiotic stimulus (GO:0071466) **(Fig 5D)**. The top biological processes among the downregulated genes include negative regulation of muscle cell differentiation (GO:0051148), oocyte morphogenesis (GO:0048601), oocyte fate determination (GO:0030716), deadenylation-independent decapping of nuclear-transcribed mRNA (GO:0031087), nuclear-transcribed mRNA catabolic process (GO:0000956), and positive regulation of meiotic cell cycle process (GO:1904146) **(Fig 5D)**. We also applied gene ontology (GO) enrichment analysis to categorize key molecular functions among the upregulated and downregulated gene populations by fold enrichment. The top molecular functions identified among the upregulated genes include extracellular matrix structural constituent conferring tensile strength (GO:0030020), oxidoreduction-driven active transmembrane transporter activity (GO:0015453), extracellular matrix structural constituent (GO:0005201), dipeptidyl-peptidase activity (GO:0008239), electron transfer activity (GO:0009055), steroid hydroxylase activity (GO:0008395), and oxidoreductase activity, acting on paired donors, with incorporation or reduction of molecular oxygen, reduced flavin or flavoprotein as one donor, and incorporation of one atom of oxygen (GO:0016712) **(Fig 5E)**. The top molecular functions identified among the downregulated genes include mRNA 3’-UTR binding (GO:0003730), cyclin-dependent protein serine/threonine kinase regulator activity (GO:0016538), ubiquitin-like protein ligase binding (GO:0044389), single-stranded RNA binding (GO:0003727), protein kinase regulator activity (GO:0019887), kinase regulator activity (GO:0019207), and mRNA binding (GO:0003729) **(Fig 5E)**. Our analysis of the RNA-seq data and our observation of increased DSBs throughout both mitotic and meiotic nuclei in the germline led us to examine whether these BBP effects might be linked to increased oxidative stress within the germline. To this end we used an oxidative stress reporter strain, *gst-4p*::*gfp; col-121(nx3)*, in which GFP expression is driven by the glutathione S-transferase 4 (*gst-4)* promoter [48]. Similar to paraquat, which is a herbicide previously reported to induce oxidative stress [49,50], we also observed a significant increase in mean GFP signal intensity in gonads from BBP-exposed worms compared to either DMSO or plain M9 buffer (*P = 0.0392; two-tailed Mann-Whitney test, 95% C.I.; **Fig 6A and 6B**). Taken together, our data suggest that exposure to 10 μM BBP, resulting in internal levels in *C*. *elegans* within the range found in different human biological samples, alters gene expression, and leads to increased oxidative stress and elevated levels of DSBs in the germline, compromising genomic integrity and the accuracy of meiotic chromosome segregation.

## Discussion

### Evidence of conserved non-monotonic dose response and turnover of BBP into MBP and MBzP metabolites in *C*. *elegans*

Here, using the highly tractable nematode *C*. *elegans*, we were able to rapidly assess the reprotoxic effects of various doses of BBP to determine the smallest dose that elicited the strongest effect. A high-throughput screening strategy using the COPAS BioSorter [27,28] revealed that from among exposures to 1, 10, 100 and 500 μM BBP, a dose of 10 μM BBP elicited the strongest effect, resulting in a 1.7-fold increase in X-chromosome nondisjunction. Analysis of germ cell apoptosis and germline chromosome morphology further indicated that a dose of 10 μM BBP is the lowest dose eliciting the strongest effects, underscoring the non-monotonic nature of BBP exposure in *C*. *elegans*. Numerous studies using human cell lines, mammalian models, and non-mammalian models have shown that EDC exposure exhibits non-monotonic effects when measuring reproductive and developmental toxicity [17]. Thus, the ability to detect non-monotonicity in our study highlights the conservation of this effect in *C*. *elegans*.

Further evidence of the conserved nature of the response to BBP exposure in *C*. *elegans* stems from our analysis of the internal levels of BBP, at 10 μM and 100 μM, and identification of its primary metabolites. In general, phthalates undergo a two-step metabolic process. The first is phase I hydrolysis (detoxification step), mediated by lipases and esterases in the intestines and parenchyma, which creates monoester phthalates. The second is phase II conjugation after further biotransformation of long-branched phthalates. In the case of BBP, metabolites are eliminated primarily in conjugated form [9]. Six metabolites of BBP were found in female Wistar rat urine following BBP administration: MBP, MBP *ω*-ox, MBzP, benzoic acid, hippuric acid, and phthalic acid [51]. Biomarker analysis in humans confirms MBP and MBzP to be the primary metabolites of BBP [8]. Furthermore, BBP has been shown to be rapidly metabolized by male Fischer-344 rats and its metabolites, MBP and MBzP, are predominately eliminated in urine though they may also pass through feces [7].

In response to 10 μM BBP exposure, we detected an internal concentration of 0.083 μg/mL of BBP, 0.104 μg/mL of MBP, and 0.282 μg/mL of MBzP in worms. Although the kinetics of BBP hydrolysis into its primary metabolites can vary by species [52], our mass spectrometry data revealed that *C*. *elegans* both rapidly and efficiently metabolize BBP, as suggested by the low levels of BBP relative to its metabolites. Epidemiological studies exploring the correlation between phthalate exposure and reproductive health issues have reported internal concentrations of BBP and its metabolites in various human biological samples. One such study included 207 women from Southwest China between 18–35 years of age who had delivered children within the past two years at the time of the study. The internal concentration of BBP detected in cord blood (at the 95^th^ percentile) was 0.089 μg/mL [18], comparable to the 0.083 μg/mL of BBP we detected in *C*. *elegans*. Their study reported an association between the presence of BBP with reduced gestational age and preterm delivery. Additionally, while fetal growth parameters seemed to be partially affected by gestational age, there were also sexual dimorphic effects of BBP, affecting primarily female infants. Another study evaluated the efficiency of using human amniotic fluid as a biomarker for detecting phthalates and their primary metabolites. Amniotic fluid was obtained from 54 pregnant women during routine amniocentesis. Of the 10 metabolites assessed, MBP was detected at a maximum concentration of 0.263 μg/mL, again comparable to the 0.104 μg/mL detected in worms [33]. Lastly, a separate study examined urine samples from 488 Spanish mothers during their first and third trimester of pregnancy. MBzP was detected at a maximum maternal urinary concentration of 0.336 μg/mL [32], comparable to the 0.282 μg/mL of MBzP detected in worms. In their study, MBzP was associated with increased femur length, a positive correlation with birth weight in boys, and a negative correlation with birth weight in girls. Taken together, the evidence of a non-monotonic dose response to BBP exposure in worms and the fact that internal levels of BBP and its primary metabolites detected in the worms are comparable to levels found in various human female biological samples underscore the relevance of using *C*. *elegans* for studying the reprotoxic effects of exposure to BBP.

### BBP-induced altered gene expression linked to increased oxidative stress and impaired genomic integrity in the *C*. *elegans* germline

Several studies support the genotoxic potential of phthalate exposure. Given the prevalence of phthalates in cosmetics, the genotoxic potential of phthalate-containing perfumes was assessed *in vitro* using human lymphoblastoid TK6 cells. Exposure to either phthalate-containing perfumes or individual phthalates, led to significant increases in DNA damage relative to negative control as assessed by comet and micronucleus assays [53]. Furthermore, with the main route of toxicant exposure from cosmetic products being dermal absorption, a human keratinocyte cell line was used as a proxy for assessing the cutaneous toxicity of phthalates. BBP exposure was shown to reduce cell viability and increase the expression of H2A.X, a marker of DSBs [54]. Exposure of *C*. *elegans* to dibutyl phthalate (DBP) resulted in p53-dependent checkpoint activation, as well as elevated meiotic DSB formation in the germline. DBP exposure, however, did not affect meiotic progression [28]. *C*. *elegans* exposure to diethylhexyl phthalate (DEHP), led to p53-dependent checkpoint activation, elevated meiotic DSB formation, and impaired meiotic progression [55]. In contrast to DBP and DEHP exposure, BBP was found to elevate DSB formation both in mitotic and meiotic germline nuclei. The different defects observed following exposure to these phthalates further highlights the need to assess the individual effects of phthalates, despite them being members of the same class of chemicals.

To better understand the potential mechanism of BBP-induced DNA damage throughout the germline, we performed RNA-seq analysis on whole worm lysates from DMSO or BBP-exposed worms. Among the 344 statistically significant differentially expressed genes, the top 8 genes by effect size included *cyp-35a3*, *cyp-35a2*, *cyp-35c1*, *ugt-22*, *hmit-1*.*1*, DH11.2, Y116A8C.9, and Y37H2A.14. The three cytochrome p450 genes, *cyp-35a3*, *cyp-35a2*, *cyp-35c1*, as well the UDP-glucuronosyltransferase gene, *ugt-22*, play a critical role in phase I and phase II xenobiotic metabolism, respectively [45]. qRT-PCR analysis from the BBP-exposed freshwater snail *Physella acuta*, also showed an increase in the expression of genes involved in phase I and phase II detoxification [45]. Similarly, increased cytochrome p450 gene expression has been detected in BBP-exposed primary fibroblasts derived from the embryos of striped field mice [56]. A recent study applying an analytical framework for multivariate mediation analysis to understand the association between various pathways and phthalate toxicity on pregnancy outcomes, found evidence for mediation through the cytochrome p450 pathway linked to decreased gestational age at delivery [57]. Among the top 8 differentially expressed genes we identified in *C*. *elegans* following BBP exposure is also *hmit-1*.*1*, which is predicted to be involved in myo-inositol transport and transmembrane transport. DBP exposure in rats, at a dose shown to cause testicular atrophy, was reported to increase levels of testicular inositol [58,59]. Myo-inositol specifically, plays a role in various metabolic pathways [46]. In fact, studies have found that myo-inositol supplementation in humans may be an effective treatment or preventive measure for symptoms related to impaired endocrine function of the ovary or preterm birth, respectively [46,60]. Given the endocrine-disrupting nature of BBP, the increased expression of a gene that plays a role in myo-inositol transport may serve as a defense mechanism against the effects of BBP exposure.

GO term analysis of our gene expression data by the biological process and molecular function categories revealed potential pathways perturbed by BBP exposure. Consistent with the upregulation of cytochrome p450 genes, xenobiotic metabolic process (GO:0006805) and cellular response to xenobiotic stimulus (GO:0071466) were among the list of upregulated biological processes. We also found upregulation of processes related to the extracellular matrix (ECM) including extracellular matrix organization (GO:0030198) and extracellular structure organization (GO:0043062). Exposures to EDCs have been reported to alter processes related to the ECM. Analysis of postnatal day 1 offspring from pregnant rats treated with the endocrine-disrupting chemical bisphenol A (BPA) showed altered levels of ECM-producing cells and ECM balancing regulators in the anterior pituitary gland [61]. Additionally, cortical organoids obtained from human embryonic stem cells showed altered cell-ECM interactions in response to DEHP exposure [62]. Future studies are required to further understand the role the ECM plays in BBP-induced reprotoxicity. The biological processes for which we observed downregulated gene expression included oocyte morphogenesis (GO:0048601) and oocyte fate determination (GO:0030716), consistent with our observation of chromosome morphology defects in the -1 oocyte at diakinesis. We also detected downregulation in the positive regulation of meiotic cell cycle process (GO:1904146), consistent with our data showing altered meiotic progression in response to BBP exposure.

Among the list of molecular functions that are altered in response to BBP exposure, we found activities broadly related to metabolic processes, ECM organization, transcription, translation, and protein ubiquitination and degradation. Of the GO molecular functions that are upregulated, oxidoreduction was of particular interest given its potential to induce DNA damage. Expression of glutathione S-transferase (*gst-4*), an enzyme involved in phase II detoxification, is activated by the SKN-1 transcription factor in response to oxidative stress [48]. Using the *gst-4p*::*gfp;col-121* reporter strain, we determined that BBP leads to increased oxidative stress in the germline. An association between BBP exposure and oxidative stress has been observed in various non-germline cells or tissues in systems including chick embryos, earthworms (*Eisenia fetida*), aquatic invertebrates, zebrafish, and human peripheral blood mononuclear cells [63–68]. Recent studies have shown the oxidative stress potential of BBP during later stages of meiosis. BBP exposure of porcine oocytes led to impaired spindle assembly and abnormal chromosome alignment, mitochondrial dysfunction marked by increased ROS, and elevated apoptosis and autophagy [23]. KEGG (Kyoto Encyclopedia of Genes and Genomes) and GO analysis from RNA-seq performed on BBP-exposed mouse oocytes at metaphase II revealed differential expression of genes playing a role in mitochondrial function, oxidoreductase, and apoptotic signaling [24]. Consistent with their RNA-seq data, they observed that BBP exposure causes mitochondrial dysfunction, oxidative stress, DNA damage, and apoptosis during mouse oocyte maturation. Our study specifically highlights BBP-induced oxidative stress at the meiotic prophase I stage, which had not been previously demonstrated.

Altogether, our data shows that at a dose of exposure resulting in internal levels comparable to what is found in human biological samples, oxidative stress is elevated in the germline, potentially underlying the significant increase in DSBs throughout the germline. We propose that the elevated DNA damage observed in BBP-exposed worms is linked to the altered meiotic progression, p53-dependent elevated germ cell apoptosis, chromosome morphology defects detected in oocytes in late diakinesis, and the increased rate of chromosome nondisjunction/aneuploidy, underscoring the reprotoxic nature of BBP.

## Materials and methods

### *C*. *elegans* lines

All *C*. *elegans* lines used in this study were maintained on NGM plates seeded with *E*.*coli* (OP50) and stored at 20°C as in [69]. Lines used include the wildtype N2 Bristol strain, *col-121(nx3)*, *Pxol-1*::*gfp; col-121(nx3)*, *cep-1;col-121(nx3)*, *rad-54*.*L/hT2;col-121(nx3)*, *gst-4p*::*gfp;col-121(nx3)* and *Ppie-1*::*gfp*::*cosa-1;col-121(nx3)*.

### Liquid culture chemical exposure

To obtain age-matched worms for chemical exposure, gravid worms were subjected to sodium hypochlorite treatment for 5 minutes with gentle agitation [70]. After a series of washes with M9 buffer, the remaining eggs were incubated in M9 overnight without bacteria, allowing eggs to hatch and arrest at the L1 stage. Arrested L1s were thoroughly washed with M9, plated on NGM plates with OP50, and incubated at 20°C until reaching the L4 stage. The day preceding chemical exposure, OP50 was inoculated in LB media and placed in a shaking incubator overnight at 37°C. On the day of the chemical exposure, L4-stage worms were resuspended in M9 with freshly cultured OP50 bacteria (OD_600_ = 24) to achieve a density of 1,200 worms per mL. BBP (Sigma Aldrich, St. Louis, MO) dissolved in DMSO at 0.1M for final concentrations of 1, 10, 100, or 500 μM and 0.1% DMSO (vehicle control) were added directly to the liquid cultures containing L4 worms and dispensed onto 24-well plates at a density of 300 worms per 250 μL. Worms were exposed for 24 hours at 20°C. Following exposure, worms were washed five times with M9 buffer and plated on NGM plates with OP50 for subsequent analysis.

### Screening with the COPAS BioSort

To establish parameters for gating adult worms with and without GFP+ embryos we used *Pxol-1*::*gfp; col-121(nx3)* worms exposed to DMSO vehicle or 100 μM of DEET [30], a positive control for X-chromosome nondisjunction. Populations of age-matched L1s, L4s, and adults were run separately through the COPAS BioSort. In between each run, the system was washed with water, 10% bleach, and cleaning solution to avoid cross contamination. Using FlowJo software, we gated for the adult population and established a threshold for GFP+ embryos in utero over background. Once the gating parameters were established, *Pxol-1*::*gfp; col-121(nx3)* worms were synchronized and exposed to DMSO (0.1%) or BBP (1, 10, 100, or 500 μM) for 24 hours as above. A total of 4,000 worms were screened per condition in 2 biological repeats. Data was analyzed using FlowJo software and reported as the fold increase over DMSO vehicle control.

### Scoring brood size, embryonic lethality, and frequency of male progeny

Age-matched worms exposed in liquid to either BBP or vehicle control (0.1% DMSO) for 24 hours, were then washed and singled onto NGM plates with OP50 bacteria and allowed to lay eggs for 24 hours. Worms were then moved every 24 hours to new NGM plates with OP50 (this was done for 3 consecutive days) and on day 4 each worm was removed and discarded from the third set of plates. Brood size was determined by scoring the total number of eggs laid over the three 24-hour windows. Embryonic lethality was determined by calculating the percentage of unhatched eggs relative to the total number of eggs laid. The frequency of male progeny was determined by calculating the percentage of adult males among the total number of worms reaching adulthood. Statistical analysis was performed using the Fisher’s exact test.

### Germ cell apoptosis

Following chemical exposure, adult hermaphrodites were incubated with 25 μg/mL of Acridine Orange in M9 with an ample amount of OP50 [31]. Foil-wrapped tubes were placed on a nutator at room temperature for 2 hours to allow for Acridine Orange to stain germ cell corpses. Worms were allowed to recover on NGM plates with OP50 (protected from light) for 20 minutes and then mounted on 1.5% agarose pads on slides with 0.01% Levamisole to slow movement. A Leica DM5000B fluorescence microscope was used to score germ cell corpses. Statistical analysis was performed using an unpaired two-tailed Mann-Whitney Test, 95% C.I.

### Immunofluorescence microscopy

Rapid cytological analysis of large numbers of DAPI-stained germlines within intact whole worms was performed by fixing worms with Carnoy’s fixative (6 parts 100% Ethanol, 2 parts chloroform, and 1-part acetic acid) followed by staining with 4’,6-diamidino-2-phenylindole (DAPI) as in [71] with slight changes. Specifically, air-dried worms were rehydrated with M9 buffer solution for 30 minutes prior to mounting coverslips with Vectashield (Vector Laboratories, Burlingame, CA) and DAPI stain solution. Slides were refrigerated for at least 2 hours to allow for DAPI to permeate into the gonads and then imaged using a Leica DM5000B fluorescence microscope.

Immunostaining of whole-mounted dissected gonads was performed as in [42], except that worms were placed on positively charged slides with 0.01% Levamisole and dissected gonads were fixed with either 1% or 4% formaldehyde, depending on the primary antibody being used. Antibodies were used at the following dilutions: rabbit anti-RAD-51 (1:10,000; Novus Biologicals (SDI); 4% fixation), guinea pig anti-pSUN-1 Ser8-pi (1:700 [35]; 1% fixation), and chicken anti-GFP (1:500; Abcam; 1% fixation). Secondary antibodies from Jackson ImmunoResearch Laboratories (West Grove, PA) used: anti-rabbit Alexa 488 (1:500), anti-guinea pig Cy3 (1:200), anti-chicken Cy3 (1:200), and anti-rabbit Cy5 (1:100).

### Quantitative spatiotemporal analysis for RAD-51 foci

Quantitative analysis of the number of RAD-51 foci per nucleus was performed for all seven zones composing the germlines of *col-121* and *rad-54*.*L;col-121* worms as described in [42]. In *col-121* worms, the average number of nuclei scored per zone (n) from at least five gonads for each condition were as follows: DMSO: zone 1 = 94, zone 2 = 142, zone 3 = 123, zone 4 = 121, zone 5 = 127, zone 6 = 121, and zone 7 = 106. BBP: zone 1 = 123, zone 2 = 145, zone 3 = 151, zone 4 = 124, zone 5 = 120, zone 6 = 110, and zone 7 = 94. In *rad-54*.*L;col-121* line, the average number of nuclei scored per zone (n) from at least six gonads for each condition were as follows: DMSO: zone 1 = 132, zone 2 = 194, zone 3 = 193, zone 4 = 179, zone 5 = 144, zone 6 = 113, and zone 7 = 81. BBP: zone 1 = 148, zone 2 = 173, zone 3 = 180, zone 4 = 173, zone 5 = 153, zone 6 = 109, and zone 7 = 78. Numbers of foci scored per nucleus are indicated in S1 Data. Statistical comparisons were performed using the two-tailed Mann-Whitney test, 95% C.I.

### Analysis of mitotic nuclear diameter

Nuclear diameters at the premeiotic tip were measured using the ruler tool in the SoftWorx 3.3.6 program (Applied Precision). Six gonads were scored per condition. Measurements per nucleus are indicated in S1 Data. Statistical comparisons between treatments were performed using the two-tailed Mann-Whitney test, 95% C.I.

### Oxidative stress assay

To assess oxidative stress, *gst-4p*::*gfp; col-121* worms were chemically exposed as described above. This reporter strain has GFP fused to the promoter of glutathione S-transferase 4, where oxidative stress induces GFP expression [48]. Following exposure, worms were washed in M9 five times, and their gonads dissected and isolated from their carcasses to minimize background autofluorescence. Dissected gonads were imaged using the Leica DM5000B fluorescence microscope and fluorescence intensities were quantified using ImageJ software. *gst-4p*::*GFP* worms treated for 1 hour with or without 50mM paraquat, a herbicide that induces oxidative stress in the germline [49,50], were used as positive and negative controls, respectively. Statistical analysis was performed using the unpaired two-tailed Mann-Whitney Test, 95% C.I.

### RNA sequencing and data analysis

Following 24-hour exposure to either 0.1% DMSO alone or BBP dissolved in DMSO, 1,200 worms per condition were washed five times with M9, then collected in 500 μl of Trizol (Invitrogen) for RNA extraction, and frozen at -80°C. This process was performed for a total of four independent biological repeats prior to extractions which were all done in the same day to reduce batch differences. RNA purification was performed using the Direct-zol RNA Miniprep Plus kit (Zymo Research) following manufacturer’s instructions. RNA concentrations, as well as the 260/280 and 260/230 ratios, were assessed using a Nanodrop (Thermo Fisher Scientific). The RNA was prepared for sequencing with the Kapa mRNA HyperPrep kit (Roche) and sequenced on a NovaSeq 6000 using an SP 2x50bp run (Illumina) by the Bauer Core Facility at Harvard University.

Data analysis was provided by the Harvard Chan Bioinformatics Core, Harvard T.H. Chan School of Public Health, Boston, MA. Reads were processed to counts through the bcbio RNA-seq pipeline implemented in bcbio-nextgen v1.2.9-8eb78b7 (https://bcbio-nextgen.readthedocs.org/en/latest/). Raw reads were examined for quality issues using FastQC (http://www.bioinformatics.babraham.ac.uk/projects/fastqc/) to ensure library generation and sequencing were suitable for further analysis. Reads were aligned to Wormbase assembly WBcel235, Annotation Version WS272, using STAR v2.6.1d [72]. Alignments were checked for evenness of coverage, rRNA content, genomic context of alignments (for example, alignments in known transcripts and introns), complexity and other quality checks using a combination of FastQC, Qualimap [73] MultiQC (https://github.com/ewels/MultiQC) and custom tools. Transcripts Per Million (TPM) measurements per isoform were generated by quasialignment using Salmon v1.6.0 [74] and transformed to counts per gene estimated by tximport [75]. Quantitating at the isoform level has been shown to produce more accurate results at the gene level. Principal component visualization of the data revealed a strong batch effect. Therefore, we applied Combat-seq from the sva package v.3.46.0 [76], which uses a negative binomial regression to model batch effects and provide adjusted data by mapping the original data to an expected distribution as if there were no batch effects. Differential expression at the gene level was called using adjusted counts with DESeq2 v1.38.3 [77]. QC and differential expression analyses were done using R programming language, v.4.2.2. For data visualization, heatmaps were generated with the pheatmap R package v1.0.12 [78] and volcano plots were generated with the EnhancedVolcano package v. 1.16.0 [79].

RNA sequencing data have been deposited in NCBI’s Gene Expression Omnibus and are accessible through GEO Series accession number GSE264361 (https://www.ncbi.nlm.nih.gov/geo/query/acc.cgi?acc=GSE264361).

### Worm lysate preparation and mass spectrometric chemical analysis

The internal concentrations of BBP were determined using gas chromatography-mass spectrometry (GC-MS) and its two primary metabolites, MBP and MBzP, were determined using LC-MS/MS. DMSO or BBP-exposed worms were washed 10 times with M9, flash frozen with minimal M9 in liquid nitrogen, and worm pellets were resuspended in lysis buffer [25 mM HEPES (pH 7.6), 5 mM EDTA, 0.5 M sucrose, 0.5% CHAPS, and 0.5% DOC (Deoxychloric acid)]. Each sample was subjected to sonication at 4°C for 30 seconds on/45 seconds off per cycle for a total of 20 cycles with a Bioruptor Plus 300 (Diagenode, Belgium). After centrifugation at 10,000 rpm for 15 min, the resulting supernatant was sent for mass spectrometric analysis. The internal concentrations of BBP, MBP, and MBzP were determined in worm lysates using isotopic dilution mass spectrometric methods. The extraction and analysis protocol for BBP and its metabolites were reported previously [28,55]. Briefly, BBP was extracted with hexane and analyzed using GC-MS (Thermo trace 1310 GC coupled with ISQ MS). The metabolites, MBP and MBzP, were enzymatically deconjugated and extracted using solid-phase extraction (SPE) before analysis using LC-MS/MS (Agilent 1100 HPLC coupled with AB Sciex API 4500 QTRAP MS). Trace levels of BBP and its metabolites were found in procedural blanks, and the values in blanks were subtracted for reporting the final concentrations in all samples (both control and dosed worms). The matrix spike test (20 ng of BBP in DMSO) was conducted for BBP to estimate the recovery of this analyte through the method, and it was 95%. For BBP metabolites, National Institute of Standards and Technology (NIST) certified standard reference materials (SRM 3672 and SRM 3673) were included in analysis. The recoveries (accuracy) of BBP metabolites from the reference materials ranged from 91 to 110%.

## Supporting information

S1 Data Raw data for analyses in main figures. (XLSX)

S1 Fig BBP exposure results in increased embryonic lethality and male progeny. Plate phenotyping data acquired from *col-121(nx3)* worms exposed to 0.1% DMSO vehicle control or 1, 10, 100, and 500 μM of BBP. Total brood size, embryonic lethality (%), and incidence of male progeny (%) was determined. Error bars represent SEM. *P = 0.0118, ****P<0.0001 by the Fisher’s exact test. N = 9–10 worms per condition. Two biological repeats.
(TIF)

S2 Fig BBP does not alter crossover designation levels. **(A)** Representative images of late pachytene nuclei from *Ppie*::*gfp*::*cosa-1;col-121(nx3)* hermaphrodites exposed to DMSO or BBP showing DAPI (blue) and GFP::COSA-1 (green). Scale bar, 5 μm. **(B)** Quantification of the mean number of GFP::COSA-1 foci per nucleus in late pachytene. P = 0.6288 by the two-tailed Mann-Whitney test, 95% C.I. N > 300 nuclei per condition. Three biological repeats.
(TIF)
