# Supplementary figures and images for: Exposure to benzyl butyl phthalate (BBP) leads to increased double-strand break formation and germline dysfunction in Caenorhabditis elegans
Source: PLoS Genet. 2024 Oct 24;20(10):e1011434. doi: 10.1371/journal.pgen.1011434 (PMC11500915; doi:10.1371/journal.pgen.1011434)

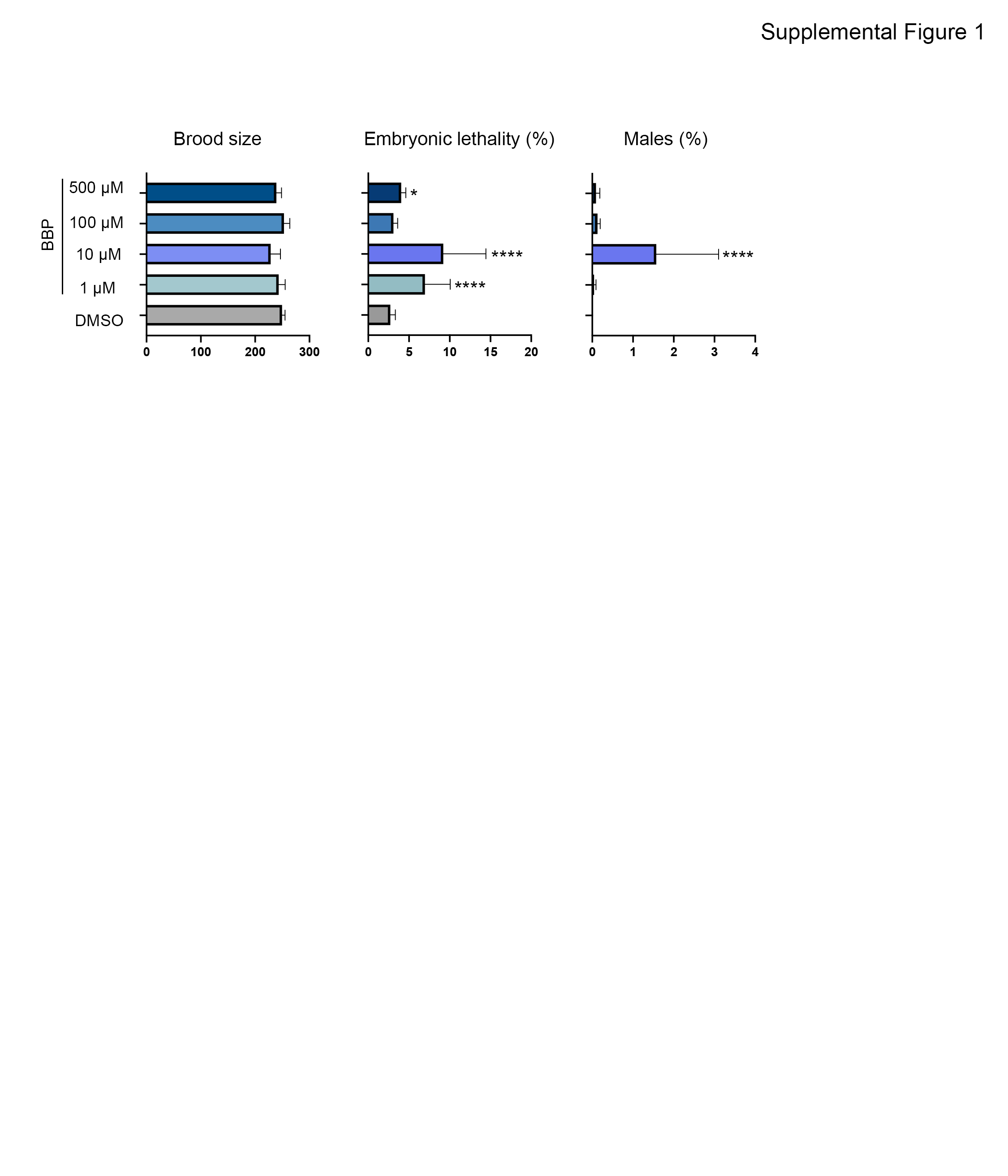

Supplement: S1 Fig — Plate phenotyping data acquired from col-121(nx3) worms exposed to 0.1% DMSO vehicle control or 1, 10, 100, and 500 μM of BBP. Total brood size, embryonic lethality (%), and incidence of male progeny (%) was determined. Error bars represent SEM. *P = 0.0118, ****P<0.0001 by the Fisher’s exact test. N = 9–10 worms per condition. Two biological repeats. (TIF) [file pgen.1011434.s002.tif]

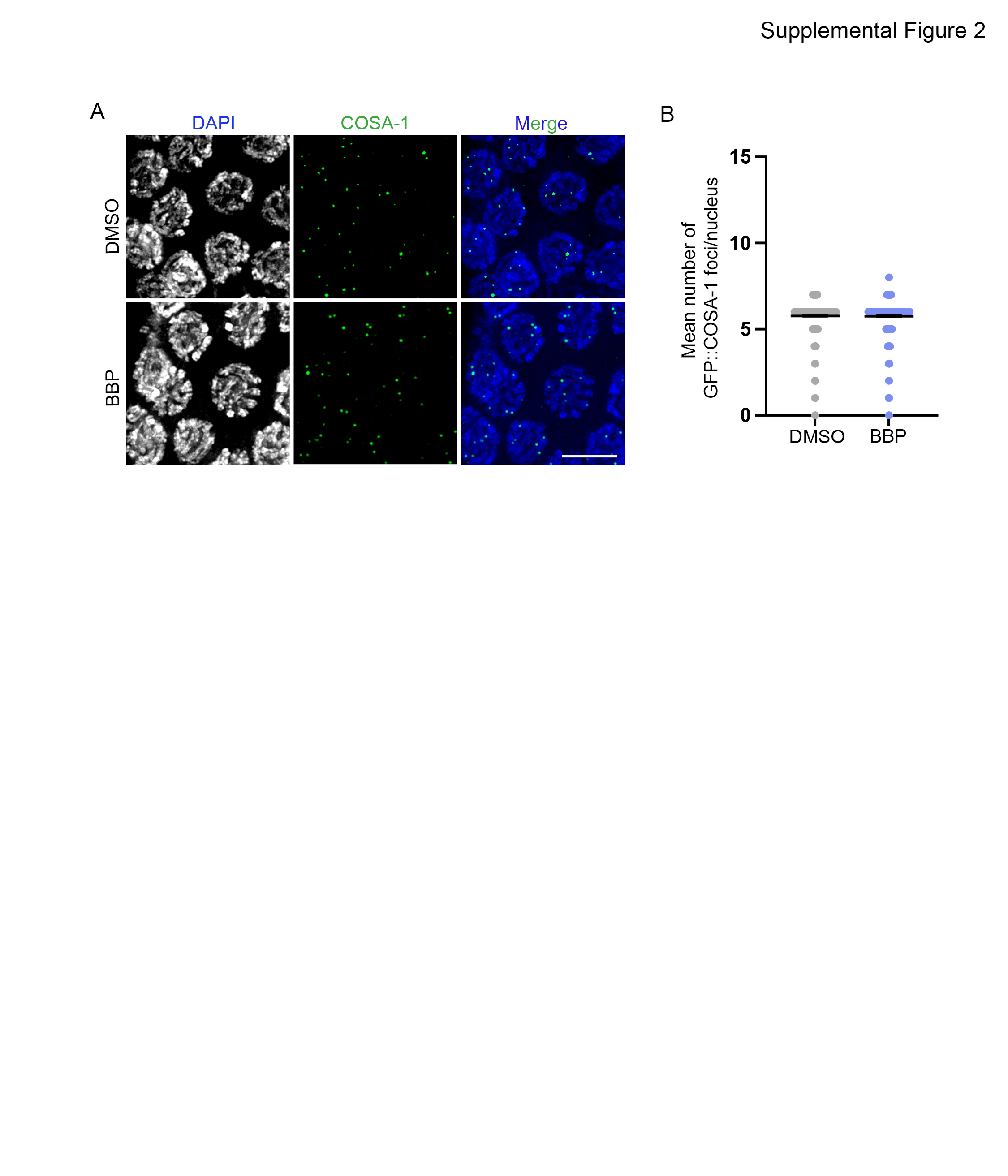

Supplement: S2 Fig — (A) Representative images of late pachytene nuclei from Ppie::gfp::cosa-1;col-121(nx3) hermaphrodites exposed to DMSO or BBP showing DAPI (blue) and GFP::COSA-1 (green). Scale bar, 5 μm. (B) Quantification of the mean number of GFP::COSA-1 foci per nucleus in late pachytene. P = 0.6288 by the two-tailed Mann-Whitney test, 95% C.I. N > 300 nuclei per condition. Three biological repeats. (TIF) [file pgen.1011434.s003.tif]
